# Non-Additive Entropy Composition Rules Connected with Finite Heat-Bath Effects

**DOI:** 10.3390/e24121769

**Published:** 2022-12-03

**Authors:** Tamás Sándor Biró

**Affiliations:** 1 Wigner Research Centre for Physics, 1121 Budapest, Hungary; biro.tamas@wigner.hu; 2 Institute for Physics, University Babeş-Bolyai, 400294 Cluj, Romania

**Keywords:** entropy, finite heat-bath, superstatistics

## Abstract

Mathematical generalizations of the additive Boltzmann–Gibbs–Shannon entropy formula have been numerous since the 1960s. In this paper we seek an interpretation of the Rényi and Tsallis q-entropy formulas single parameter in terms of physical properties of a finite capacity heat-bath and fluctuations of temperature. Ideal gases of non-interacting particles are used as a demonstrating example.

## 1. Introduction

Entropy is a great tool in thermodynamics and statistical physics. Conceptualized originally by Clausius [1] as a state descriptor, that distinguishes it from heat, it became a basic principle for statistical and informatics calculations. Its classical form, the Boltzmann entropy has a wide use [2,3,4,5]. Nevertheless, mostly in mathematical approaches to informatics, its generalizations occurred: altered, non-logarithmic formulas between the probability of a given sate and the total entropy of the system [6,7,8,9,10].

Still, the classical logarithmic formula is of widest use, having a number of properties that make it destined to be a convex measure of information and probability. The most known generalization is due to Alfred Renyi [6], who constructed a formula being also additive for factorizing joint probability, but abandoned the logarithm as the sole function with this property. There is a parameter, occurring as a power of probability, denoted by *q* or α. The classical formula emerges in the q→1 limit, formally.

As interesting as the Renyi entropy is, its form is not an expectation value. The *q*-entropy as an expectation value was suggested by C. Tsallis [11,12,13], as that form is not additive for factorizing joint probabilities (or being additive for correlated, non-factorizing probabilities). Other and further generalizations, naming more parameters, were also suggested formulated in terms of leading order corrections to the Boltzmann formula in the thermodynamical limit or just utilizing more parameters for possible deformations of the original formula [9,14,15,16,17,18,19]. Properties of generalized entropy formulas were intensely studied, for two selected examples with respect to the Tsallis entropy see [20,21].

In the present paper another viewpoint is presented: (i) first we identify deviations from the classical logarithmic formula derived from deviations from additivity; (ii) then we demonstrate how phase space finiteness effects cause corrections to the additivity of entropy in coincidence with the factorization of probability in a microcanonical approach; (iii) and finally we ask the question of which modified entropy can be the most additive one in this respect. More closely, a general group entropy [22] is considered following non-addition rules and a limit of its infinite repetitions on small amounts is considered as an asymptotic rule of composition [23]. Associative rules form group operations, therefore a logarithm of the formal group can be derived from a general composition law, which is then additive. This will be the content of the next section.

There were decade-long discussions about the physical (or statistical) meaning of the parameter *q*, the first non-universal parameter occurring in generalized entropy formulas. It may be bound to the sort of the system under discussion, to its material properties, but at the same time it occurs generally in a given class of statistical systems, including informatics, statistical mechanics, dynamics at the edge of chaos, and complex random networks. A few approaches in the quest of uncovering physical mechanisms determining the value of *q* in particular cases in which the present author was involved, are in Refs. [23,24,25,26,27]. Similar studies by others are copiously cited in review books, cf. [12,24].

Following this, a physical interpretation of the parameter *q* will be established connected to the finite heat capacity of an environment [28] and to possible fluctuations in phase space dimensionality. The latter is akin to the superstatistical approach [29,30,31,32,33]. A balance between physical factors reducing and increasing the value of *q* may ensure the classical q=1 case, however, in a general setting it is not provided. One is then tempted to restore additivity at best—since classical thermodynamics is based on this property. The attempt is made by using a logarithm of the formal group of entropy composition instead of the original entropy, S→K(S), and deriving again the associated $q_{K}$ parameter. Then $q_{k}=1$ generates a differential equation for the K(S) function, and due to that, a new composition rule.

Finally some examples will be discussed and families of K(S) forms will be established. The Boltzmann, Renyi, Tsallis entropies are all special cases, and they represent physical extremes in terms of the heat container capacity and the relative size of superstatistical fluctuations.

## 2. Logarithm of the Formal Group

It is worth starting our mathematical considerations with the composition law of entropy, or any other real valued physical quantity, when contacting and combining two systems to a bigger, unified one. Abandoning additivity, which ensures the co-extensivity properties of entropy and other extensive quantities, constructed also as expectation values, further composition rules are considered for non-extensive statistical mechanics [12]. The Abe-Tsallis composition law,

(1) $$S_{12}\;=\;S_{1}+S_{2}+\left(q-1\right)S_{1}S_{2},$$

is a particular case for a more general one, described by a two-variable function, x⊕y=h(x,y). In such composition rules the entropies, $S_{i}$ (i = 1, 2, or 12) can be any state functions, $S(E,N,X_{1},\ldots )$ and assumed to be valid in general, both for equilibrium and non-equilibrium entropies. Here we do not address such questions, just look for the consequences of adopting non-additive rules for the entropy. The value of the parameter *q* is usually in the open interval (0,2), in measured cases very often close but not equal to q=1. Formally, however, it can be any real number, even negative. Certainly for q<1, the entropies cannot be too large in order to avoid a negative composite result. In thermodynamics we deal mostly with large systems; thus, the requirement of associativity is natural:

(2) h(h(x,y),z)=h(x,h(y,z)).

Having the third law in mind, on the other hand, zero entropy is a valid value, and its addition must be trivial:

(3) h(x,0)=x,

and similarly h(0,y)=y. These two requirements already circumvent composition as a group operation. The question of inverse building remains only nontrivial.

Here, the logarithm of the formal group is helpful. It can be shown that, from the associativity Equation (2), it follows the existence of a monotonous and hence invertible mapping, which maps the general rule to the addition:

(4) K(h(x,y))=K(x)+K(y).

The function K(z) is the formal logarithm; it can be constructed asymptotically from the rule h(x,y) as follows. Imagine we compose from *x* to x⊕y=h(x,y) by adding small amounts, Δy, a number of time, *N*, so that NΔy=y. Then, in a general step, one proceeds as

(5) $$x_{n+1}\;=\;x_{n}⊕\Delta y\;=\;h\left(x_{n},\Delta y\right)\;=\;h\left(x_{n},0\right)\;+\;\Delta y\;\frac{\partial h}{\partial y}\left(x_{n},0\right)\;+\;ldots$$

The index *n* in this change is additive, while *x* and *y* are not. Seeking for a continuous limit in the variable t=n/N between zero and one, we arrive at

(6) $$\int_{x(0)}^{x(1)}\frac{dx}{\frac{\partial h}{\partial y}\left(x,0\right)}\;=\;\int_{0}^{1}ydt\;=\;y.$$

Here x(0)=x and x(1)=x⊕y. Denoting the primitive function of the above integral by K(z), our result reads as

(7) K(x⊕y)−K(x)=y.

This form breaks the symmetry between *x* and *y*. We remedy this problem by re-defining Δy=K(y)/N, i.e., taking steps by the additive quantity K(y). By this we obtain the K-additivity

(8) K(x⊕y)=K(x)+K(y).

This is the sought mapping to additivity; therefore, K(x) is called the formal logarithm. We note by passing this point that due to the continuous limit in the above derivation, the new rule is only asymptotic:

(9) $$h_{\infty }\left(x,y\right)\;=\;K^{-1}\left(K(x)+K(y)\right)$$

does not always coincide with the starting rule, h(x,y). Such asymptotic rules, however, build attractors among all composition rules. The result Equation (6) can also be obtained by taking the partial derivative of Equation (4) at y=0, using h(x,0)=x and integrating. The asymptotic rule is a reconstruction of the composition rule from its first derivative at a very small (zero) second argument.

Here, we mention examples. The Tsallis-Abe rule, h(x,y)=x+y+axy with a=q−1, leads to the formal logarithm $K\left(x\right)=\frac{1}{a}\ln\left(1+ax\right)$ and does not change in the asymptotics: $h_{\infty }\left(x,y\right)=x+y+axy$. A more general rule, using a general function of the product of the composables, h(x,y)=x+y+f(xy) on the other hand leads back to the Tsallis-Abe rule with $a=f^{\prime }\left(0\right)$. Triviality requires f(0)=0, of course. The properties K(0)=0 and $K^{\prime }\left(0\right)=1$ also do hold.

Lesser-known, more complex rules can also be investigated. For example, h(x,y)=(x+y)A(xy)+B(xy) results in a logarithm of a rational function for K(x). Instead of listing more and more examples, however, let us close this section with a more general comment. Once we change the simple additivity to K-additivity, equivalent to the use of an associative composition rule, the entropy formula in terms of the probability also changes.

Considering an ensemble in the Gibbs sense, the state *i* is realized $W_{i}$ times, while altogether, $W=\sum_{i}W_{i}$ instances are investigated. The probability of being in state *i* approaches the $p_{i}=W_{i}/W$ ratio. Since the individual contribution to entropy would be $-\ln p_{i}$ in the classical approach, a composition of *W* such instances from which the *i*-th is repeated $W_{i}$ times shall be constructed by K-additivity. The logarithm of the probability being additive for factorizing joint probabilities, a non-additive entropy can be constructed by the inverse function of the formal logarithm:

(10) $$S_{\mathrm{tot},\mathrm{non}-\mathrm{add}}\;=\;\sum_{i}W_{i}K^{-1}\left(-\ln p_{i}\right).$$

In this way the generalized entropy formula belongs to an ensemble average value (or expectation value):

(11) $$K^{-1}\left(S\right)\;=\;\sum_{i}p_{i}K^{-1}\left(-\ln p_{i}\right).$$

This formula may need a little more explanation. Since we replaced the original additivity assumption with K-additivity, the additive quantities, reflected in the $\ln(1/p_{i})$ formula which is additive for factorizing probabilities, must be a K-function of the non-additive ones. The above Equations (10) and (11) are for the non-additive quantities; therefore, the inverse function, $K^{-1}$ is used on the additive log.

With the example of the Tsallis–Abe composition law, we have $K\left(S\right)=\frac{1}{a}\ln\left(1+aS\right)$ and $K^{-1}\left(z\right)=\left(e^{az}-1\right)/a$. This delivers the Tsallis entropy,

(12) $$S_{T}=K^{-1}\left(S\right)\;=\;\frac{1}{a}\sum_{i}\left(p_{i}^{1-a}-p_{i}\right),$$

with q=1−a as the non-additive, but expectation value-like construction, with the corresponding Rényi entropy,

(13) $$S_{R}=K\left(S_{T}\right)=\;=\;\frac{1}{a}\ln\left(\sum_{i}p_{i}^{1-a}\right),$$

as the version additive for factorizing probabilities, but not being an expectation value (ensemble average).

Consequently, equilibrium distributions when maximizing the entropy or its monotonous function, K(S), deliver corresponding solutions of

(14) $$\frac{\partial S}{\partial p_{i}}\;=\;\alpha +\beta \epsilon_{i},$$

while keeping an average energy fixed. Both for the Rényi and Tsallis q-entropy the resulting canonical distribution becomes a cut power law in the individual energy, $\epsilon_{i}$:

(15) $$p_{i}\;=\;\frac{1}{Z}{\left(1+a\frac{\epsilon_{i}}{T}\right)}^{-1/a}.$$

In the a→0 (q→1) limit the Boltzmann-Gibbs exponential emerges. The factor *Z* ensures the normalization, $\sum_{i}p_{i}=1$.

## 3. *q* Parameter in the Boltzmannian Approach

At a first glance, it seems arbitrary which composition rule, and consequently which formal logarithm, is to be used in our models. However, the parameters occurring in a modification of the entropy addition law need some connection to physical reality. In this section we first extend the familiar textbook derivation of the exponential canonical distribution by going a step further in the thermodynamical limit expansion and then compare the result with the cut power-law canonical distribution. This gives rise to a possible physical interpretation of the parameter *q*.

Following the classical argumentation, there is a factor in the probability of a subsystem having energy ϵ out of the total *E* occurring as a ration of corresponding phase space volumes:

(16) $$\rho \left(\epsilon \right)\;=\;\langle \frac{\Omega (E-\epsilon )}{\Omega (E)}\rangle .$$

Here, the averaging is over parallel ensemble copies of the same system, allowing for microscopical fluctuations in parameters beyond the total energy, *E*, like particle numbers, charges, etc. The occupied phase space volumes are connected to the entropy, $\Omega \left(E\right)=e^{S(E)}$. Expanding the expression Equation (16) up to first order in ϵ≪E one arrives at the well known canonical factor

(17) $$\rho_{1}\left(\epsilon \right)\;=\;\langle e^{-\epsilon S^{\prime }\left(E\right)}\rangle \;=\;e^{-\epsilon \langle S^{\prime }\left(E\right)\rangle }\;=\;\frac{1}{Z}e^{-\epsilon /T}.$$

Here *Z* is a normalization factor ensuring $\int_{0}^{E}\rho \left(\epsilon \right)d\epsilon \;=\;E$. The temperature is interpreted as $1/T=\langle S^{\prime }\left(E\right)\rangle$. The information about the environment (heat bath) is comprised into this single parameter, traditionally.

Now we look at the consequences of going one step further, i.e., performing an $O(\epsilon^{2})$ expansion:

(18) $$\rho_{2}\left(\epsilon \right)\;=\;\langle e^{-\epsilon S^{\prime }\left(E\right)+\epsilon^{2}S^{\prime \prime }\left(E\right)/2}\rangle \;=\;1-\epsilon \langle S^{\prime }\left(E\right)\rangle +\frac{1}{2}\epsilon^{2}\langle S^{\prime \prime }\left(E\right)+S^{\prime }{\left(E\right)}^{2}\rangle .$$

This result is to be compared with the canonical distribution following from the Rényi and Tsallis entropy, to the Tsallis–Pareto distribution [12,34] to the same order

(19) $${\left(1+\left(q-1\right)\frac{\epsilon }{T}\right)}^{-\frac{1}{q-1}}\;=\;1-\frac{\epsilon }{T}+\frac{q}{2}\;\frac{\epsilon^{2}}{T^{2}}.$$

Term by term comparison between Equations (18) and (19) interprets the parameters *T* and *q* in the Tsallis–Pareto distribution as being

(20) $$\frac{1}{T}\;=\;\langle S^{\prime }\left(E\right)\rangle ,\;\frac{q}{T^{2}}\;=\;\langle S^{\prime \prime }\left(E\right)\rangle +\langle S^{\prime }{\left(E\right)}^{2}\rangle .$$

Denoting $S^{\prime }\left(E\right)=\beta$ as a fluctuating quantity, one uses its variance in the above interpretation of *q*, besides the derivative of the temperature dT/dE=1/C, with *C* being the total capacity of the heat bath in the formula $\langle S^{\prime \prime (E)}\rangle =\frac{d}{dE}\frac{1}{T}=-1/CT^{2}$:

(21) $$q\;=\;1-\frac{1}{C}+T^{2}\Delta \beta^{2}.$$

This result allows for *q* values both smaller and larger than one; the q=1 remaining a special choice. Indeed, textbooks [35] suggest that the q=1 is the only possible choice and then conclude that $T\Delta \beta =1/\sqrt{C}$ argues for the ”one over square root law” for energy fluctuations. This argumentation is, however, misleading: the physical situation and size of the heat bath actually present must determine the value of the parameter *q*.

## 4. Optimal Restoration of Additivity

In the present section we shall optimize the choice of K(S) instead of *S* in estimating the phase space volumes above in order to achieve $q_{K}=1$. This requirement leads to a differential equation restricting the function K(S). Since according to the Tsallis-Abe composition law q=1 is the additive case, seeking for $q_{K}=1$ we call restoration of additivity. This is the best result possible, keeping in mind that the Tsallis-Pareto distribution is also an approximation in case of finite heat bathes, although one term improved beyond the traditional Boltzmann–Gibbs exponential.

The canonical statistical factor in this case transmutes to

(22) $$\rho_{K}\;=\;\langle e^{K(S(E-\epsilon ))-K(S(E))}\rangle .$$

Expanding up to $(\epsilon^{2})$ terms, as in the previous section, with the assumption that the K(S) function is universal, i.e., independent from the energy stored in the heat bath, we obtain

(23) $$\rho_{K}\;=\;1-\epsilon \langle S^{\prime }\rangle K^{\prime }\;+\;\frac{\epsilon^{2}}{2}\left[\langle S^{\prime \prime }\rangle K^{\prime }+\langle S^{\prime \;2}\rangle \left(K^{\prime \;2}+K^{\prime \prime }\right)\right]\;+\;\ldots$$

Comparing this with a Tsallis–Pareto distribution of the same approximation, we obtain another temperature and different variance parameters, $T_{K}$ and *q*:

(24) $$\begin{array}{ccc} \frac{1}{T_{K}}\; & = & \;\langle S^{\prime }\rangle \;K^{\prime }\\ q_{K}\; & = & \;\langle S^{\prime \prime }\rangle \frac{T^{2}}{K^{\prime }}+\langle S^{\prime \;2}\rangle \left(1+\frac{K^{\prime \prime }}{K^{\prime \;2}}\right). \end{array}$$

The requirement, $q_{K}=1$, singles out a K(S) formal logarithm for the entropy composition rule, which optimizes to the subleading order $O(\epsilon^{2})$ in general. This results in a simple, solvable differential equation for $F=1/K^{\prime }$:

(25) $$q_{K}\;=\;-\frac{1}{C}\;F+\left(1+T^{2}\Delta \beta^{2}\right)\left(1-F^{\prime }\right)\;=\;1.$$

Here, we again used the fact that $\langle S^{\prime }\rangle =1/T$ and $\langle S^{\prime \prime }\rangle =-1/CT^{2}$. Ordering this equation to $F^{\prime }$ one obtains

(26) $$F^{\prime }\;+\;\frac{1/C}{1+T^{2}\Delta \beta^{2}}\;F\;=\;\frac{T^{2}\Delta \beta^{2}}{1+T^{2}\Delta \beta^{2}}.$$

Such an equation can be solved by quadrature even for complicated functions of the entropy.

The simplest physical system is an ideal gas: in this case, the heat capacity is independent of the total entropy, so 1/C is a constant. On the other hand, we may also assume that the relative variance in temperature, comprised in the term TΔβ=Δβ/〈β〉, is also a constant with respect to the total entropy. In this simplest case, it is straightforward to obtain the optimal formal logarithm, K(S) from Equation (26).

Here we present the solution, which contains two parameters:

(27) $$K\left(S\right)\;=\;\frac{1}{\mu }\;\ln\left(1+\frac{\mu }{\lambda }\left(e^{\lambda S}-1\right)\right).$$

This ansatz is a “to and back” construction in a $K\left(S\right)=h_{\mu }\left(h_{\lambda }^{-1}\left(S\right)\right)$ form with $h_{\mu }\left(x\right)=\frac{1}{\mu }\ln\left(1+\mu x\right)$ being the formal logarithm associated to the Tsallis–Abe composition rule. The reciprocal inverse of the first derivative of K(S) is obtained as

(28) $$F\left(S\right)\;=\;\frac{1}{K^{\prime }\left(S\right)}\;=\;\frac{\mu }{\lambda }+\left(1-\frac{\mu }{\lambda }\right)e^{-\lambda S}$$

satisfying the F(0)=1 condition. Substituting this function and its first derivative into Equation (26) we conclude that $F^{\prime }+\lambda F=\mu$ with

(29) $$\begin{array}{ccc} \mu \; & = & \;\frac{T^{2}\Delta \beta^{2}}{1+T^{2}\Delta \beta^{2}},\\ \lambda \; & = & \;\frac{1/C}{1+T^{2}\Delta \beta^{2}}. \end{array}$$

It is interesting to check some limits of this expression. For μ≪λ or in the μ=0 case, corresponding to zero fluctuations in the thermodynamical temperature, one obtains

(30) $$K\left(S\right)\;=\;\frac{1}{\lambda }\left(e^{\lambda S}-1\right)\;=\;C\left(e^{S/C}-1\right).$$

This generates a non-additive entropy formula

(31) $$K^{-1}\left(S\right)\;=\;C\;\sum_{i}p_{i}\ln\left(1-\frac{1}{C}\ln p_{i}\right).$$

The corresponding canonical distribution is a complicated expression involving Lambert’s function.

In the other extreme, λ=0, meaning the presence of an infinite heat capacity (ideal) heat bath, we arrive at

(32) $$K\left(S\right)\;=\;\frac{1}{\mu }\ln\left(1+\mu S\right).$$

In this case the non-additive entropy formula delivers a Tsallis entropy, cf. Equations (11) and (12), and the canonical distribution is a Tsallis–Pareto distribution.

It is most intriguing that the choice μ=λ, i.e., stating that the temperature fluctuations are exactly following the inverse square root law, TΔβ=1/sqrtC; and therefore, μ=λ=1/(C+1), always the Boltzmann–Gibbs formula emerges:

(33) K(S)=S,

and *S* itself is additive.

One realizes that the balance between ensemble fluctuations and the finiteness of the heat bath determines which is the optimally additive entropy formula. It is convenient to use the formal logarithm K(S) instead of *S* as an additive quantity. Still, the traditional balance derived from q=1 (λ=μ) is not always given in physical situations. Some might find it strange that the parameter *q* depends on the heat capacity controlling the reservoir. Here the relation is with the same system’s heat capacity. Analogously, a much simpler correspondence is true for a fixed volume of photon gas, where C=3S, since the equation of state is given by $S∼E^{3/4}V^{1/4}$, stemming from $E/V∼T^{4}$ and $S/V∼T^{3}$.

## 5. Fluctuations in Phase Space Dimension

One of the most prominent cases when fluctuations are “external”, occurring due to the way of collecting data and not stemming from the finiteness of the heat bath, is the study of single particle energy spectra in high energy collisions. Hadronization makes *n* particles, event by event a different number, while the total energy shared by them is approximately constant. This situation is opposite to the energy fluctuations with a fixed number of particles (atoms).

In this case the phase space to be filled by individual energies has a fluctuating dimension, while the total energy determining the microcanonical hypersurface is fixed. Then, depending on the actual probability of making *n* hadrons in a single collision event, $P_{n}$, the summed distribution of single particle energy, frequently measured by the transverse momentum for very energetic particles, will differ from the traditionally expected exponential or Gaussian. In order to dwell on this problem, let us first review how the microcanonical phase space is calculated at a given total energy, *E*, and how dimensions for *n* particles move in some spatial dimensions.

Phase space is over momenta. Individual energies are functions of momenta according to the corresponding dispersion relation. A number of such relations look like a power of the absolute value, so they can be comprised into an $L_{p}$-norm:

(34) $${\left(\sum_{i=1}^{n}{\left|p_{i}\right|}^{p}\right)}^{1/p}\leq R\left(E\right)$$

with $p_{i}$ individual momentum components, altogether n-dimension in phase space and *E* total energy. The R(E) function also reflects the dispersion relation between energy and momenta. For a one dimensional jet $E=|\vec{p}|$, here simply R(E)=E and the $L_{1}$ norm is to be used. For nonrelativistic ideal gases $E=|\vec{p}{|}^{2}/2m$, therefore, $R\left(E\right)=\sqrt{2mE}$ and the $L_{2}$ norm is used.

For extreme relativistic particles p=1, and R(E)=E measures the volume satisfying

(35) $$\sum_{i=1}^{n}\left|p_{i}\right|\;\leq \;E.$$

The general formula reads as

(36) $$\Omega_{n}^{\left(p\right)}\left(R\right)\;=\;\frac{\Gamma {\left(1/p+1\right)}^{n}}{\Gamma (n/p+1)}\;{\left(2R\right)}^{n}.$$

Originally Dirichlet obtained this formula in a french publication [36]. More contemporary popularizations are due to Smith and Vamanamurthy [37] from 1989 and Xianfu Wang [38] from 2005. Wikipedia also has an entry on this formula [39] and a recursive proof in few lines can be obtained from [40]. A microcanonical constrained hypersurface is the derivative of the above volume formula against the total energy

(37) $$G_{n}^{\left(p\right)}\left(E\right)\;=\;\frac{d}{dE}\;\Omega_{n}^{\left(p\right)}\left(R\left(E\right)\right).$$

Meanwhile the surface is the derivative against *R*.

We define a ratio of volumes, and consider the p=1 case, related to relativistic particles in a 1-dimensional jet:

(38) $$r_{n}^{\left(1\right)}\;=\;\frac{\Omega_{1}^{\left(1\right)}\left(\epsilon \right)\Omega_{n-1}^{\left(1\right)}\left(E-\epsilon \right)}{\Omega_{n}^{\left(1\right)}\left(E\right)}\;=\;n\frac{\epsilon }{E}{\left(1-\frac{\epsilon }{E}\right)}^{n-1}.$$

For normalization we use the pure environmental factor, the above ratio without the single particle factor:

(39) $$\rho_{n}^{\left(1\right)}\;=\;\frac{\Omega_{n-1}^{\left(1\right)}\left(E-\epsilon \right)}{\Omega_{n}^{\left(1\right)}\left(E\right)}\;=\;\frac{n}{2E}{\left(1-\frac{\epsilon }{E}\right)}^{n-1}.$$

This $\rho_{n}^{\left(1\right)}\left(\epsilon ,E\right)$ is normalized over an integral in the single-particle phase space. This means an integral over *p* between −E and +E, while ϵ=|p| with the absolute value:

(40) $$\int_{-E}^{+E}\rho_{n}^{\left(1\right)}dp\;=\;\frac{n}{2E}\;\int_{-E}^{+E}{\left(1-\frac{\left|p\right|}{E}\right)}^{n-1}\;dp\;=\;1.$$

Due to the |p| absolute value expression, this integral is twice of that between 0 and *E*, whence the factor 2 in the denominator becomes cancelled.

Once its integral is normalized to 1, also the mixture

(41) $$\rho^{\left(1\right)}\left(\epsilon ;E\right)\;=\;\sum_{n=0}^{\infty }P_{n}\rho_{n}^{\left(1\right)}\left(\epsilon \right)$$

is normalized to 1, provided that the $\sum_{n}P_{n}=1$ sum is also normalized.

Finally, note that the ratio of volumes and energy shells in the 1-dimensional, relativistic case are simply related:

(42) $$G_{n}^{\left(p\right)}\left(E\right)\;=\;\frac{d}{dE}\Omega_{n}^{\left(p\right)}\left(R\left(E\right)\right)\;=\;\Omega_{n}^{\left(p\right)}\left(R\left(E\right)\right)\;\frac{d}{dE}\ln\left(R^{n}\right),$$

in the p=1 (relativistic) $L_{1}$-norm delivers due to R(E)=E

(43) $$G_{n}^{\left(1\right)}\left(E\right)\;=\;\frac{n}{E}\;\Omega_{n}^{\left(1\right)}\left(E\right).$$

So we have $G_{1}^{\left(1\right)}\left(\epsilon \right)=\frac{1}{\epsilon }\Omega_{1}^{\left(1\right)}=2$ and

(44) $$g_{n}^{\left(1\right)}\left(\epsilon ;E\right)\;=\;\frac{G_{1}^{\left(1\right)}\left(\epsilon \right)G_{n-1}^{\left(1\right)}\left(E-\epsilon \right)}{G_{n}^{\left(1\right)}\left(E\right)}\;=\;\frac{n-1}{E}{\left(1-\frac{\epsilon }{E}\right)}^{n-2}.$$

Finally, we have

(45) $$r_{n}^{\left(1\right)}\left(\epsilon ;E\right)\;=\;\epsilon \;g_{n+1}^{\left(1\right)}\left(\epsilon ;E\right).$$

In this case the microcanonical ratio, $g_{n+1}^{\left(1\right)}$ is normalized to 1 for an integral over ϵ between 0 and *E*.

For ideal gases, one considers $S=\ln c_{n}+n\ln E$, delivering

(46) $$\frac{1}{T}\;=\;\frac{\langle n\rangle }{E};\;\mathrm{and}\;\frac{q}{T^{2}}\;=\;\frac{\langle n(n-1)\rangle }{E^{2}}.$$

Here, *q* is actually the measure of non-Poissonity,

(47) $$q\;=\;1-\frac{1}{\langle n\rangle }+\frac{\Delta n^{2}}{{\langle n\rangle }^{2}}.$$

For the negative binomial distribution (NBD) q=1+1/(k+1), for the Poissonian exactly q=1. In hadronization statistics the Tsallis–Pareto distribution extracted from transverse momentum distributions and the multiplicity fluctuations event by event go hand in hand [41,42,43]. Such distributions may be a consequence of dynamical random processes, too, as it is investigated in the framework of the Local Growth Global Reset (LGGR) model recently on the master equation level [44], or earlier in the framework of the generalization of Boltzmann’s kinetic approach and the related H theorem [45,46,47].

## 6. Conclusions

In conclusion, we investigated the physical background for applying non-additive entropy in three steps: (i) we reviewed associative composition rules and the derivation of their asymptotic version valid in the thermodynamical limit, (ii) we have optimized which formal logarithm of the entropy, K(S), is to be used in phase space occupation probability arguments, including but not restricted to the case for Tsallis entropy, and finally, (iii) we reviewed phase space ratios in high energy jets as an application for superstatistical fluctuation in the dimensionality of phase space volumes. The coupling of these three aspects in a particular chain concludes that even ideal gases in a finite heat bath environment and away from thermal equilibrium can show a certain ambiguity, best removed by using non-additive entropy formulas.

To show an example, a certain ambiguity for estimating the heat capacity from maximizing the mutual entropy between observed subsystem and heat bath is discussed in detail for ideal gases in the Appendix A. We demonstrate in Appendix B that using K(S) instead of *S* indeed clears the mismatch between statistical informatics—postulating mutual entropy maximum in equilibrium—and thermodynamics, obtaining the heat capacity of a subsystem independently of the heat bath, i.e., using the Universal Thermostat Independence (UTI) principle.

## Data Availability

Not applicable.

## Appendix A. Ideal Gas with Finite Heat-Bath

First, we describe how a finite heat capacity heat bath influences the heat capacity of an observed subsystem. The effect stems from maximizing the mutual entropy instead of the subsystem’s entropy alone.

We consider ideal gases, occupying phase space volumes according to the N-Ball in $L_{p}$ norm picture. *N* is the number of degrees of freedom, or particles, while dimensionality factorizes. For one-dimensional extreme relativistic particles, the radius is R(E)=E and one uses the diamond shape, $L_{1}$-norm. For traditional, nonrelativistic particles the radius is $R\left(E\right)=\sqrt{2mE}$ and one uses the spherical, $L_{2}$-norm.

In both cases the entropy is given as

(A1) S=ln(a(N))+kNlnE.

We have k=1 for one-dimensional jets, and k=3/2 for traditional ideal gases in 3 dimensions. The first derivative wrsp the energy defines the β=1/T variable:

(A2) $$\beta \;\equiv \;S^{\prime }\left(E\right)\;=\;k\frac{N}{E}\;=\;1/T.$$

The second derivative defines implicitly the heat capacity:

(A3) $$C\;\equiv \;{\left.\frac{dQ}{dT}\right|}_{V}\;=\;\frac{dE}{dT}\;=\;\frac{dE}{d(1/\beta )}\;=\;kN$$

with

(A4) $$S^{\prime \prime }\left(E\right)\;=\;-\frac{1}{CT^{2}}\;=\;-\frac{\beta^{2}}{kN}.$$

For a subsystem connected with another system (may be called reservoir if large enough) not the individual, but the mutual entropy maximum describes the most probable energy for a subsystem. We have $I_{12}=S\left(E_{1},N_{1}\right)+S\left(E_{2},N_{2}\right)-S\left(E_{1}+E_{2},N_{1}+N_{2}\right)$ and when fixing $E_{2}$ and all *N*-s from that the first derivative,

(A5) $$\frac{\partial I_{12}}{\partial E_{1}}\;=\;k\frac{N_{1}}{E_{1}}\;-\;k\frac{N_{1}+N_{2}}{E_{1}+E_{2}},$$

and the second derivative against $E_{1}$ is given by

(A6) $$\frac{\partial^{2}I_{12}}{\partial E_{1}^{2}}\;=\;-k\frac{N_{1}}{E_{1}^{2}}\;+\;k\frac{N_{1}+N_{2}}{{\left(E_{1}+E_{2}\right)}^{2}}.$$

Due to $\rho \left(E_{1}\right)∼e^{I_{12}}$, the most probable energy value for the subsystem is obtained from the vanishing of the first derivative of $I_{12}$. This occurs at $\beta_{1}=\beta_{12}$, according to the definition of β and Equation (A5). At this point from the second derivative, an effective, intercorrelated heat capacity for the subsystem appears. We have

(A7) $${\left.\frac{\partial^{2}I_{12}}{\partial E_{1}^{2}}\right|}_{\max}\;=\;-k\beta^{2}\frac{N_{2}}{N_{1}\left(N_{1}+N_{2}\right)},$$

and from that the effective heat capacity

(A8) $$C_{1,I_{12}=max}\;=\;\frac{C_{1}}{C_{2}}\left(C_{1}+C_{2}\right)\;=\;C_{1}+\frac{C_{1}^{2}}{C_{2}}.$$

Exactly the correction $C_{1}^{2}/C_{2}$ is the effect of a finite heat bath reservoir which vanishes in the thermodynamical limit, $C_{2}\to \infty$.

A similar result can be derived when fixing the total energy, $E=E_{1}+E_{2}$, and then looking for the most probable subsystem energy, $E_{1}$. We get

(A9) $$C_{1,I_{12}=max}\;=\;C_{1}-\frac{C_{1}^{2}}{C}.$$

In this case the total system has fixed parameters (energy, heat capacity). Again, in the thermodynamical limit the correction vanishes.

## Appendix B. Universal Thermostat Independence

The above corrections, in one case positive, in another negative, make the concept of heat capacity ambiguous. In order to avoid this discrepancy, we can follow two strategies: (i) ignore the problem and restrict to the infinite capacity reservoir limit, or (ii) compensate for this leading effect near to the maximal probability. Choosing the second strategy is equivalent with admitting that the original additive concept of mutual entropy has to be generalized.

Following the second option, we consider the maximum of the mutual K-entropy instead of the original entropy, and the additive quantity associated to a possibly non-additive entropy, K(S), is constructed in a way that the corresponding heat capacity appears infinite.

It is easy to achieve by choosing K(S) accordingly. All usual quantities, temperature, heat capacity acquire a *K* index by doing so and we obtain

(A10) $$\begin{array}{ccc} \frac{1}{T_{k}}\; & = & \;\frac{\partial }{\partial E}K\left(S\right)\;=\;K^{\prime }\left(S\right)\frac{\partial S}{\partial E}\;=\;\frac{K^{\prime }}{T}\;\\ -\frac{1}{C_{K}T_{K}^{2}}\; & = & \;\frac{\partial }{\partial E}\frac{1}{T_{K}}\;=\;\frac{1}{T^{2}}\left(-K^{\prime \prime }+{\left(K^{\prime }\right)}^{2}\frac{1}{C}\right). \end{array}$$

Solving it for the *K*-capacity of heat, we have

(A11) $$\frac{1}{C_{K}}\;=\;-\frac{K^{\prime \prime }}{{\left(K^{\prime }\right)}^{2}}\;+\;\frac{1}{C}.$$

The universal thermostat independence requires that $1/C_{K}=0$, which is a second order differential equation for K(S), quoted as the UTI principle in [28,48],

(A12) $$\frac{K^{\prime \prime }\left(S\right)}{{\left(K^{\prime }\left(S\right)\right)}^{2}}\;=\;\frac{1}{C}.$$

With the boundary conditions K(0)=0 (for the sake of keeping the third law of thermodynamics) and $K^{\prime }\left(0\right)=1$ (just keeping the Boltzmann constant at its original value $k_{B}=1$), we arrive at a solution for a constant *C*, independent of *S*, as being

(A13) $$K\left(S\right)\;=\;-C\ln\left(1-S/C\right).$$

Applying now *K*-additivity with this formula, we require zero mutual K-entropy reflecting total independence of the energy states of reservoir and subsystem,

(A14) $$K\left(S_{1}\right)+K\left(S_{2}\right)-K\left(S_{12}\right)\;=\;0,$$

we derive the Tsallis–Abe composition law. From Equations (A13) and (A14), one writes

(A15) $$-C\ln\left(1-S_{1}/C\right)-C\ln\left(1-S_{2}/C\right)\;=\;-C\ln\left(1-S_{12}/C\right).$$

From here, a product rule follows,

(A16) $$\left(1-\frac{S_{1}}{C}\right)\cdot \left(1-\frac{S_{2}}{C}\right)\;=\;\left(1-\frac{S_{12}}{C}\right),$$

which in turn simplifies to

(A17) $$S_{12}\;=\;S_{1}+S_{2}-\frac{1}{C}S_{1}S_{2}.$$

Comparing this with the Tsallis–Abe rule Equation (1) one obtains q=1−1/C. This is already a part of the result presented in Equation (21). For the total result, previously known temperature fluctuation can already be counted for, which may or may not under- or overcompensate this effect.
